# Outcomes of recurrent stroke in patients with atrial fibrillation according to presumed etiology

**DOI:** 10.1055/s-0043-1769124

**Published:** 2023-06-19

**Authors:** Bruno Bacellar Pedreira, Korilyn Sauser Zachrison, Aneesh Singhal, Zhiyu Yan, Jamary Oliveira-Filho, Lee H. Schwamm

**Affiliations:** 1 Harvard Medical School, Massachusetts General Hospital, Department of Neurology, Boston, United States.; 2 Universidade Federal da Bahia, Hospital Universitario Professor Edgard Santos, Programa de Pós-Graduação em Ciências da Saúde, Salvador BA, Brazil.; 3 Harvard Medical School, Massachusetts General Hospital and Boston, Department of Emergency Medicine, Boston, United States.

**Keywords:** Ischemic Stroke, Atrial Fibrillation, Embolic Stroke, Anticoagulants, AVC Isquêmico, Fibrilação Atrial, AVC Embólico, Anticoagulantes

## Abstract

**Background** Atrial fibrillation (AF) is a potent risk factor for stroke. The presence of competing etiologies can modify disease outcomes and demand different treatment strategies.

**Objectives** The primary purpose of the study was to examine the differences in outcomes for patients with AF admitted with a recurrent stroke, stratified according to the presumed etiology of the stroke.

**Methods** We analyzed AF patients admitted for a recurrent ischemic stroke in an academic comprehensive stroke center. Recurrent strokes were categorized as “Cardioembolic”, meaning AF without any competing mechanism, versus “Undetermined” etiology due to competing mechanisms. We used logistic regression to test the association between recurrent stroke etiology and favorable outcome (discharge home), after accounting for important covariates.

**Results** We included 230 patients, with a mean age 76.9 (SD ± 11.3), 52.2% male, median National Institute of Health Stroke Scale (NIHSS) score of 7 (IQR 2–16). Patients with cardioembolic stroke (65.2%) had higher median NIHSS 8.5 (3–18) versus 3 (1–8) and were more likely to be treated with reperfusion therapies. The favorable outcome was reached by 64 patients (27.8%), and in-hospital mortality was 15.2% overall. After adjustment, there was no difference in outcome between patients with cardioembolic versus undetermined stroke etiology (odds ratio for discharge home: 1.41; 95% CI: 0.65–3.15).

**Conclusions** In this single-center sample of AF patients with history of stroke, there was no difference in discharge outcomes between those with cardioembolic and those with undetermined stroke etiology. This question warrants examination in larger samples to better understand the importance of the stroke mechanism and secondary prophylaxis.

## Resumo

**Antecedentes** Fibrilação atrial (FA) é um fator de risco importante para AVC. A presença de mecanismos concorrentes para o AVC pode modificar o desfecho e demandar estratégias de tratamento diferentes.

**Objetivos** O objetivo primário do estudo foi examinar diferenças no desfecho de pacientes com FA admitidos por um AVC recorrente, sendo estratificados de acordo com a etiologia presumida do AVC.

**Métodos** Nós analisamos pacientes com FA admitidos por conta de AVC recorrente em um centro acadêmico terciário de AVC. Os casos de AVC recorrentes foram classificados como “Cardioembólicos”, sendo FA sem outros mecanismos alternativos, versus aqueles de etiologia “Indeterminada” por conta de mecanismos concorrentes. Foi usada regressão logística para testar a associação entre a etiologia do AVC recorrente e desfecho favorável (alta direto para casa) após controle para covariáveis importantes.

**Resultados** Nós incluímos 230 pacientes, com uma idade média 76,9 anos (DP ± 11.3), 52.2% homens, com um escore mediano do National Institute of Health Stroke Scale (NIHSS) de 7 (IIQ 2–16). Pacientes com AVC cardioembólicos (65,2%) tiveram um escore de NIHSS mediano mais alto 8,5 (3–18) versus 3 (1–8), e com maior chance de tratamento com terapias de reperfusão. O desfecho favorável ocorreu em 64 pacientes (27,8%) e a mortalidade institucional foi de 15,2% no total. Após ajustes, não encontramos diferença no desfecho entre pacientes com AVC cardioembólico versus AVC de etiologia indeterminada (odds ratio para alta para casa: 1,41; 95% IC: 0,65–3,15).

**Conclusões** Nessa amostra de pacientes com FA e história de AVC recorrente de centro único, não houve diferença no desfecho de alta entre aqueles com AVC cardioembólico e aqueles com etiologia indeterminada. Essa questão deve ser examinada em amostras maiores para melhor compreender a importância do mecanismo do AVC e a profilaxia secundária.

## INTRODUCTION

Atrial fibrillation (AF) is a potent risk factor for stroke, associated with up to a five-fold increase in ischemic stroke risk. 1 Globally, the estimated number of individuals with atrial fibrillation and flutter was 37.6 million in 2017. 2 It has an age-dependent prevalence of up to 3% in the adult population over 40 years old, and several studies suggest that the prevalence of AF is rising. 3 4 5 6 7 8 Ischemic stroke patients with AF are at high risk of stroke recurrence. This risk can be dramatically reduced by long-term anticoagulation therapy soon after the presenting event. However, stroke in these patients is not necessarily cardioembolic; 9 nearly a third of strokes in patients with AF can have a noncardioembolic mechanism. 10 11

In a metanalysis comparing oral anticoagulants versus control/placebo or antiplatelet agents in noncardioembolic stroke patients there was no benefit of anticoagulation therapy to prevent death, recurrent stroke or myocardial infarction (MI), and an increased risk of major bleed. 12 Furthermore, two trials tested direct oral anticoagulants (DOAC) versus antiplatelet agents in patients with an embolic neuroimaging phenotype but no documented embolic source, and again no benefit of anticoagulation was shown. 13 14

Many believe that secondary prophylaxis should be tailored according to the presumed etiologic mechanism. Anticoagulation therapy may not prevent stroke recurrence in noncardioembolic strokes. 15 Moreover, some patients, especially those with small vessel disease, could have an increased risk of intracranial bleeding. 16 17 On the other hand, patients with AF and previous history of stroke also have an increased risk of a future, possibly disabling, cardioembolic ischemic stroke. 16 18 While usual care for patients with ischemic stroke and atrial fibrillation is to start oral anticoagulants, the presence of competing etiologies may modify disease outcomes and, therefore, require different treatment strategies.

The primary purpose of this study was to analyze outcomes for AF patients admitted with acute recurrent stroke, stratified according to the presumed etiology of the stroke. As a secondary objective, we examined whether prestroke antithrombotic use was associated with stroke subtype in this population.

We hypothesized that patients with previous AF and a recurrent cardioembolic stroke would have a worse prognosis, evidenced by lower likelihood of being discharged home—when compared to in-hospital mortality or discharge to a facility.

## METHODS

This study was conducted among patients admitted with a recurrent ischemic stroke and a previous diagnosis of AF or paroxysmal AF. We used data from our stroke registry that included all consecutive patients with stroke from our urban academic comprehensive stroke center from January 2015 to December 2020. We did not include patients for whom AF was identified only at the index admission (i.e., without past history). We also excluded patients for whom information of the stroke etiologic mechanism was missing. The data collection project has been reviewed and approved by the MGH Institutional Review Board and, given the retrospective nature of this study, and there being a minimal risk to the subjects, informed consent was waived.

The trial of org 10172 in acute stroke treatment (TOAST) classification 19 was used for the registry and, in our study, patients were further categorized according to the presumed stroke mechanism: definite “Cardioembolic”, meaning AF without a competing mechanism; versus “Undetermined”, under which we grouped all other etiologies—given the possibility of competing mechanisms besides the AF. The primary outcome of interest was favorable (discharged home vs not). The study did not involve therapeutic intervention. All patients were treated at the discretion of the stroke team, following validated guidelines and institutional protocols.

Descriptive statistics are presented as mean/standard deviations (SD) for normally distributed continuous variables, median/interquartile range for non-normally distributed continuous and ordinal variables, and absolute numbers and proportion (%) for categorical variables. The distribution was analyzed by visual inspection of the histogram and with the Shapiro-Wilk test. To compare characteristics between etiologies, continuous variables were compared using the Student t test or the Mann-Whitney U test as appropriate, while categorical variables were compared using the Fisher exact test.

Logistic regression models were used to test the association between the presumed stroke etiology and outcomes. We tested three predefined models in which variables were chosen a priori, based on existing literature and clinical experience: model 1 adjusted for age, sex, anticoagulation status, receipt of intravenous alteplase (IV tPA), receipt of mechanical thrombectomy (MT) and admission National Institute of Health Stroke Scale (NIHSS). Model 2 included the aforementioned variables, as well as patients' comorbidities (such as hypertension, diabetes, renal failure etc.). Finally, model 3 included only comorbidities potentially associated with the binary outcome in univariable analyses ( *p* < 0.2). For each model, the association between stroke etiology and each outcome was considered significant if the *p* -value < 0.05.

To examine the association of antithrombotic use and stroke etiology we used logistic regression adjusting for age, sex, NIHSS and patients' comorbidities (hypertension, diabetes, dyslipidemia, obesity, heart failure, renal failure).

Analyses were performed using the R statistical (R Foundation for Statistical Computing, Vienna, Austria) software.

## RESULTS

A total of 1,141 consecutive patients were admitted with a recurrent ischemic stroke or transient ischemic attack (TIA) in the period of January 2015 to December 2020, of which 230 met our inclusion criteria and were part of the analysis ( Figure 1 ). A comparison between included and excluded patients is shown in Table 4 . For the 230 included patients, the mean age was 76.9 years old (SD ± 11.3), and 120 (52.2%) were male. The majority of patients were white (81.7%), and the median NIHSS score was 7 (interquartile range [IQR]: 2–16).

**Abbreviation:**
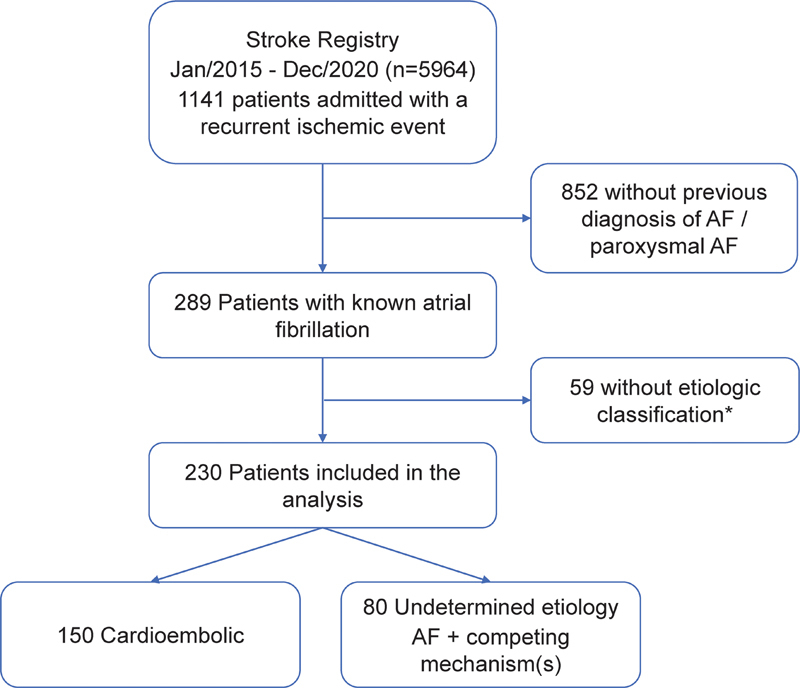
AF, atrial fibrillation. **Note:** *Comparison with included patients is shown in Table 4 . **Figure 1** Patient inclusion flow diagram.

From the total of 230 patients included in this study, 150 (65.2%) had a cardioembolic stroke (AF without other competing mechanism). Compared to patients with stroke of undetermined mechanism, cardioembolic stroke patients had more severe strokes with median NIHSS scores, 3 (1–8) versus 8.5 (3–18) respectively, and were more commonly treated with reperfusion therapies: intravenous tissue plasminogen activator (IV tPA), 8.0 versus 2.5%, and mechanical thrombectomy (MT) 14.0 versus 3.8%, respectively ( Table 1 ).

**Table 1:** Patients admitted with a recurrent stroke and known prior atrial fibrillation

	All patients (n = 230)	Cardioembolic (n = 150)	Undetermined (n = 80)	*P* -value
Age				0.192
Mean (SD)	76.9 (11.3)	77.4 (11.8)	76.1 (10.4)	
Median (IQR)	78 (69–86)	78 (69–87)	77 (68–84)	
Male sex, n (%)	120 (52.2)	72 (48.0)	48 (60.0)	0.097
Race / ethnicity, n (%)				0.041
Hispanic	11 (4.8)	9 (6.0)	2 (2.5)	
Non-H Asian	10 (4.4)	9 (6.0)	1 (1.3)	
Non-H black	16 (7.0)	13 (8.7)	3 (3.8)	
Non-H white	188 (81.7)	114 (76.0)	74 (92.5)	
Unknown	5 (2.2)	5 (3.3)	0	
Diabetes, n (%)	76 (33.0)	50 (33.3)	26 (32.5)	1.000
Hypertension, n (%)	181 (78.7)	115 (76.7)	66 (82.5)	0.398
Dyslipidemia, n (%)	146 (63.5)	89 (59.3)	57 (71.3)	0.085
Smoking, n (%)	20 (8.7)	12 (8.0)	8 (10.0)	0.628
Obesity / overweight, n (%)	60 (26.1)	43 (28.7)	17 (21.3)	0.270
Heart failure, n (%)	56 (24.4)	40 (26.7)	16 (20.0)	0.333
CAD / Prior MI, n (%)	82 (35.7)	51 (34.0)	31 (38.8)	0.474
Prosthetic Heart Valve, n (%)	4 (1.7)	3 (2.0)	1 (1.3)	1.000
Renal failure, n (%)	50 (21.7)	32 (21.3)	18 (22.5)	0.868
Antithrombotic use, n (%)				0.027
Anticoagulant	133 (57.8)	81 (54.0)	52 (65.0)	
Antiplatelet only	72 (31.3)	47 (31.3)	25 (31.3)	
Not on antithrombotics	25 (10.9)	22 (14.7)	3 (3.8)	
CHADS _2_ , median (IQR)	4 (3–5)	4 (3–5)	4 (3–5)	0.632
NIHSS, median (IQR)	7 (2–16)	8.5 (3–18)	3 (1–8)	< 0.001
Reperfusion therapy, n (%)				
IV tPA	14 (6.1)	12 (8.0)	2 (2.5)	0.022
IA treatment	24 (10.4)	21 (14.0)	3 (3.8)	0.031
Favorable outcome (discharge home)	64 (27.8)	37 (24.7)	27 (33.8)	0.165

**Abbreviations:** CAD, coronary arterial disease; IA, intra-arterial; IQR, interquartile range; CHADS, Congestive heart failure, Hypertension, Age, Diabetes, prior Stroke (stroke risk prediction); IV tPA, intravenous tissue plasminogen activator; MI, myocardial infarction; NIHSS, National Institute of Health Stroke Scale; SD, standard deviation.

There were 64 patients (27.8%) with a favorable outcome (discharged home after hospital admission), and in-hospital mortality was 15.2%. In bivariate analyses, age and admission NIHSS were associated with an unfavorable outcome ( Table 2 ).

**Table 2:** Predictive factors for favorable outcome, (discharge home)

Variable		Unadjusted OR	95% CI	Adjusted OR	95% CI
Etiology	Undetermined				
	Cardioembolic	0.64	(0.36–1.17)	1.41	(0.65–3.15)
Antithrombotic use	Anticoagulant (reference)				
	Antiplatelet only	0.72	(0.37–1.36)	0.73	(0.31–1.68)
	Not in use	0.41	(0.11–1.17)	0.62	(0.11–2.77)
Age (5 years)		0.81	(0.71–0.93)	0.85	(0.72–1.01)
Male sex		1.64	(0.91–2.97)	1.33	(0.60–2.98)
IV tPA		1.04	(0.28–3.24)	6.29	(1.11–35.56)
IA treatment		0.34	(0.08–1.03)	5.45	(0.66–46.66)
Admission NIHSS (4 points)		0.39	(0.27–0.54)	0.30	(0.18–0.45)

**Abbreviations:** CI, confidence interval; IA, intra-arterial; IV tPA, intravenous tissue plasminogen activator; NIHSS, National Institute of Health Stroke Scale; OR, odds ratio.

After adjustment for important covariates (model 1), there was no association between cardioembolic stroke etiology and favorable outcome (adjusted odds ratio [aOR]: 1.41, 95% confidence interval [CI] = 0.65–3.15). The patient characteristics that were associated with being discharged home in adjusted analysis were: IV tPA (aOR 6.29, 95% CI = 1.11–35.56) and admission NIHSS (aOR for each 4 points increase was 0.30, 95% CI = 0.18–0.45) ( Table 2 ).

The additional analysis as per prespecified multivariate models including risk factors (model 2) and variables potentially associated with the binary outcome in univariate analyses ( *p* < 0.2) (model 3), yielded similar results, with no significant association between the presumed etiology and outcome.

When comparing patients who were admitted with acute stroke previously using anticoagulants, antiplatelets, or neither ( Table 3 ), the likelihood of a cardioembolic etiology (no competing mechanism) was higher when none of the two agents were used when compared to anticoagulant use (OR = 4.71; 95% CI = 1.53–20.59).Furthermore, there was no difference between antiplatelet and anticoagulant use (OR = 1.21; 95% CI = 0.67–2.21) for the likelihood of a cardioembolic etiology.

**Table 3:** Predictive factors for cardioembolic etiology (no competing mechanism(s)

Variable		Unadjusted OR	95% CI	Adjusted OR	95% CI
Antithrombotic use					
Anticoagulant (reference)	Antiplatelet only	1.21	(0.67–2.21)	1.30	(0.65–2.67)
	Not in use	4.71	(1.53–20.59)	4.71	(1.12–33.74)
Age (5 years)		1.05	(0.93–1.18)	0.98	(0.84–1.14)
Male sex		0.62	(0.35–1.06)	0.63	(0.32–1.23)
Hypertension		0.70	(0.34–1.37)	0.55	(0.21–1.37)
Diabetes		1.04	(0.59–1.87)	0.88	(0.44–1.77)
Dyslipidemia		0.59	(0.32–1.05)	0.78	(0.36–1.68)
Obesity		1.49	(0.79–2.89)	1.69	(0.79–3.73)
Heart failure		1.45	(0.77–2.87)	1.67	(0.75–3.88)
Renal failure		0.93	(0.49–1.82)	0.95	(0.45–2.07)
Admission NIHSS (4 points)		1.45	(1.22–1.76)	1.40	(1.16–1.73)

**Abbreviations:** CI, confidence interval; OR, odds ratio; NIHSS, National Institute of Health Stroke Scale.

## DISCUSSION

This is a real-world, retrospective study using a cohort of patients with stroke treated in a single-center. In the analysis of patients with previous AF and admission for a recurrent stroke, it was found that prior anticoagulant use was associated with stroke etiology; furthermore, we did not find an association between stroke etiology and favorable poststroke outcome.

The most common serious arrythmia type is AF, and it accounts for the majority of cardioembolic stroke cases. Such cases are known to be associated with worse outcomes relative to other etiologies. 20 Furthermore, it has been shown that the NIHSS score predicts the likelihood of recovery after stroke. 21 Accordingly, in our study, cardioembolic strokes were associated with a higher admission NIHSS (median 8.5 [IQR: 3–18] vs. 3 [1–8], *p* < 0.001). However, we did not find an association of stroke etiology and likelihood of favorable outcome. It is possible that this population, composed of AF patients with prior strokes, was at a greater risk for unfavorable outcomes (occurred in 72% of patients). Another possibility is that the relationship between stroke type and outcome was confounded by the greater frequency of reperfusion therapies in cardioembolic stroke patients (8.0 vs. 2.5% for IVtPA, and 14.0 vs. 3.8% for IA treatment). This finding is similar to another study, which found that history of AF was not associated with worse outcomes when compared with other cardioembolic strokes. 22

Investigators in that study also drew attention to the lost opportunity of anticoagulation therapy, especially in such a high-risk population. Consistent with data from stroke registries that show an unjustifiable underuse of anticoagulation in atrial fibrillation patients, 23 24 25 in our sample, more than 40% of patients were not on anticoagulation medication. We found that the use of this type of therapy was lower in patients with cardioembolic etiology of their recurrent stroke, supporting this need for optimization of secondary prophylaxis.

This study has some limitations. First, given its retrospective nature and the use of secondary data, we were unable to include patients for whom we did not have information of the stroke mechanism, which may have introduced bias. However, it is reassuring that the study population had similar baseline characteristics and clinical outcomes when compared to the excluded population, suggesting representativeness ( Table 4 ).

**Table 4:** Comparison with not-included patients (due to missing data)

		All patients (n = 289)	Included patients (n = 230)	Not included (n = 59)	*P* -value
Age	Mean (SD)	77.3 (11.3)	76.9 (11.3)	78.6 (10.9)	0.304
	Median (IQR)	78 (69–85)	78 (69–86)	81 (72.5–85)	0.296
Male sex, n (%)		156 (54)	120 (52.2)	36 (61)	0.244
Race / ethnicity, n (%)	Hispanic	11 (3.8)	11 (4.8)	0	0.444
	Non-H Asian	12 (4.2)	10 (4.4)	2 (3.4)	
	Non-H black	21 (7.3)	16 (7)	5 (8.5)	
	Non-H White	238 (82.4)	188 (81.7)	50 (84.8)	
	Unknown	7 (2.4)	5 (2.2)	2 (3.4)	
Diabetes, n (%)		92 (31.8)	76 (33)	16 (27.1)	0.436
Hypertension, n (%)		229 (79.2)	181 (78.7)	48 (81.4)	0.722
Dyslipidemia, n (%)		174 (60.2)	146 (63.5)	28 (47.5)	0.036
Smoking, n (%)		22 (7.6)	20 (8.7)	2 (3.4)	0.269
Obesity/overweight, n (%)		64 (22.2)	60 (26.1)	4 (6.8)	0.001
Heart failure, n (%)		70 (24.2)	56 (24.4)	14 (23.7)	1
CAD / Prior MI, n (%)		103 (35.6)	82 (35.7)	21 (35.6)	1
Prosthetic heart valve, n (%)		4 (1.4)	4 (1.7)	0	0.585
Renal failure, n (%)		59 (20.4)	50 (21.7)	9 (15.3)	0.365
Antithrombotic use, n (%)	Anticoagulant	168 (58.1)	133 (57.8)	35 (59.3)	0.167
	Antiplatelet only	85 (29.4)	72 (31.3)	13 (22)	
	Not on antithrombotics	36 (12.5)	25 (10.9)	11 (18.6)	
CHADS _2_ , median (IQR)		4 (3–5)	4 (3–5)	4 (3–5)	0.957
NIHSS, median (IQR)		6 (2–16)	5 (2–14.25)	7 (2–16)	0.502
Reperfusion therapy, n (%)	IV tPA	16 (5.5)	14 (6.1)	2 (3.4)	0.539
	EVT	24 (8.3)	24 (10.4)	0	0.006
Favorable outcome (discharge home)		83 (28.7)	64 (27.8)	19 (32.2)	0.521

**Abbreviations:** CAD, coronary arterial disease; EVT, Endovascular thrombectomy; IQR, interquartile range; IV tPA, intravenous tissue plasminogen activator; MI, myocardial infarction; NIHSS, National Institute of Health Stroke Scale; SD, standard deviation.

Second, this was a single center study in a university-based setting, which may not generalize to community-based stroke centers. Third, the classification of the stroke mechanisms was made by the treating team as part of the clinical practice; these classifications may have greater interrater variation than if a validated formal classification algorithm was used. We were unable to calculate a kappa score; however, in our study the patients were reclassified as “cardioembolic” or “undetermined” (with other possible competing etiologies) and the ‘cardioembolic’ subtype appears to have the highest interrater agreement (> 90%). 26 Furthermore, this attribution reflects real-world practice and is representative of patients for whom treatment decisions will be made.

While we were unable to compare long-term functional outcomes between groups due to the nature of our registry data, previous studies have used discharge destination as a valid measure of poststroke patient outcome. 27 28 29

In conclusion, in this single-center comprehensive sample of patients with history of previous AF and recurrent stroke, we found no difference in outcome between those with cardioembolic versus undetermined stroke etiology, however this could be due to a type 2 error. Given the limitations, our study cannot be interpreted as conclusive. With the increasing detection of AF due to the availability of monitoring devices and aging of the general population, this question should be examined in larger samples to better understand secondary prophylaxis for stroke.
